# Editorial: Mitochondria, metabolism and cardiovascular diseases

**DOI:** 10.3389/fcvm.2022.996739

**Published:** 2022-08-19

**Authors:** Jun-ichiro Koga, Xinghui Sun, Masuko Ushio-Fukai

**Affiliations:** ^1^Second Department of Internal Medicine, University of Occupational and Environmental Health, Japan, Kitakyushu, Japan; ^2^Department of Biochemistry, University of Nebraska - Lincoln, Lincoln, NE, United States; ^3^Department of Medicine (Cardiology), Vascular Biology Center, Medical College of Georgia at Augusta University, Augusta, GA, United States

**Keywords:** mitochondria, atherosclerosis, heart failure, metabolism, reactive oxygen species

Mitochondria are intracellular organelle playing central roles in intracellular energy metabolism and reactive oxygen species (ROS) production. Recently, much attention has been focused on the role of mitochondria in the mechanisms of cardiovascular and metabolic diseases such as atherosclerosis, heart failure, hypertension, and diabetes. Mitochondrial metabolism, such as ATP production *via* β-oxidation, TCA cycle to generate metabolites, and mitochondrial electron transport chain to generate ROS, is essential for normal cell function. Of note, vascular endothelial cells mainly depend on glycolysis to produce ATP in normal physiological conditions. Although ROS at the physiological level are important for cell signaling, excess ROS derived from dysfunctional mitochondria promote cardiovascular diseases (1). In addition, mitochondrial autophagy (mitophagy) plays an important role in mitochondrial quality control by removing damaged mitochondria (2). Mitochondrial dynamics regulated by the balance of mitochondrial fission and fusion is important for maintaining mitochondrial function and health. Emerging evidence suggests that mitochondrial dysfunction, aberrations in mitochondrial metabolism, ROS levels and dynamics, and impaired mitophagy are interconnected, which contributes to the pathophysiology of cardiovascular diseases.

This Research Topic highlighted three original papers and three review articles in this area. Shosha et al. examined the metabolic function and mitochondrial structure in the retinas of control and diabetic mice. They demonstrated mitochondria fragmentation in the retina of middle-aged diabetic Akita mice, which was associated with a decrease in glycolysis and expression of 6-Phosphofructo-2-Kinase/Fructose-2,6-Biphosphatase 3 (PFKFB3), a rate-limiting enzyme of glycolysis, in the retina. However, these changes were not observed in old Akita mice at 10 months of age. As the authors have mentioned, the role of age-specific suppression of glycolysis in the pathobiology of diabetic retinopathy needs to be further examined. Mitochondrial respiratory function determined by oxygen consumption rate (OCR) measurement showed no significant differences between the Akita and control mice. However, basal respiratory activity was decreased in Akita mice under glucose-free conditions, suggesting that the metabolic stress induced by glucose deprivation and subsequent glycolysis inhibition can unmask mitochondrial dysfunction in the diabetic retinas. Future studies are needed to elucidate mechanisms underlying the difference in mitochondrial respiration under glucose or glucose-deprived conditions.

Ma et al. reported the role of iron in the mechanisms of atherosclerosis. Authors have observed increased iron content in the aorta of apolipoprotein-E^−/−^ (ApoE^−/−^) mice, accompanied by increased expression of transferrin receptor 1, ferritin, iron regulatory protein 1, iron regulatory protein 2, and heme oxygenase 1, all of which are related to iron metabolism. In addition, they found that ApoE^−/−^ mice had an increased expression of aortic ICAM1, VCAM1, LOX-1, Gpx4, and CD36, as well as increased levels of ROS in the blood. Importantly, deferoxamine, an iron chelator, abrogated atherosclerotic plaque formation and suppressed the induction of iron metabolisms-related molecules. The authors concluded that the increased uptake of iron and its accumulation in the aortic wall at least partially promote the development of atherosclerosis in ApoE^−/−^ mice, likely through iron-mediated production of ROS. It is reported that mitochondria play important roles in intracellular iron metabolisms. For example, the final step of heme biosynthesis occurs in the mitochondrial matrix (3). The importance of iron metabolism in the cardiovascular systems is largely unknown. Thus, further studies are warranted to develop anti-atherosclerosis therapies targeting iron metabolism.

Dikalov et al. reported a novel, ^15^N- and deuterium-enriched spin probe ^15^N-CAT1H, a highly sensitive and site-specific probe for extracellular superoxide. In combination with ^14^N-mitoTEMPO, phagocytic NADPH oxidase activity and mitochondrial superoxide were visualized in immune cells isolated from the spleen. Using these probes, the authors demonstrated the coupling of phagocytic NADPH oxidase activity and mitochondrial superoxide production. In splenic immune cells isolated from angiotensin II-infused mice, basal superoxide level was increased. The complex III inhibitor, mitochondrial uncoupler, and NADPH oxidase activator further increased superoxide levels in both extracellular space and mitochondria, suggesting angiotensin II-induced superoxide production by the cross-talk between NADPH oxidase and mitochondria.

The three reviews summarize up-to-date information about mitochondria's role in cardiovascular diseases. Riascos-Bernal et al. described atypical cadherin FAT1. They have reported that FAT1 is an unpredicted negative regulator of mitochondrial function, including mitochondrial metabolism, and suppresses smooth muscle cell proliferation after vascular injury. In addition, FAT1 interacts with the electron transport chain complexes and suppresses mitochondrial respiration. As a result of this action, DNA synthesis and proliferation of smooth muscle cells are suppressed, indicating that FAT1 is a potential therapeutic target for mitochondria-dependent vascular disease. Zeng et al. summarized a review focusing on the role of mitochondria in the process of vascular calcification. First, they briefly summarized the mechanisms of vascular calcification. Then, they discussed the role of mitochondria in vascular calcification and related mechanisms. For example, mitochondria are involved in intracellular calcium homeostasis and cell death, including apoptosis, which is considered relevant to the pathogenesis of vascular calcification. The article also stated that the accumulation of mitochondrial damage and inhibition of mitophagy, a mitochondrial removal mechanism, can lead to mitochondrial dysfunction and smooth muscle cell calcification. Lastly, they discussed future research directions and the main challenges. Uchikado et al. summarized the topic focused on mitochondrial dynamics and cardiovascular diseases. Recent evidence supports the hypothesis that mitochondrial morphological changes, including fission and fusion, play an important role in mitochondrial quality control mechanisms (4). Impairment of these processes is related to various cardiovascular senescence/diseases (5).

This Research Topic supports the notion that mitochondria have emerged as central factors in various aspects of cardiovascular diseases. Although this multifaceted nature of mitochondria can be an obstacle in therapeutic development, understanding and identifying key molecules involved in mitochondrial dysfunction will lead to developing novel effective therapies and targets required for the treatment of various cardiovascular diseases.

## Author contributions

All authors have contributed conceptual design, drafted the manuscript, and approved the final version for publication.

## Conflict of interest

The authors declare that the research was conducted in the absence of any commercial or financial relationships that could be construed as a potential conflict of interest.

## Publisher's note

All claims expressed in this article are solely those of the authors and do not necessarily represent those of their affiliated organizations, or those of the publisher, the editors and the reviewers. Any product that may be evaluated in this article, or claim that may be made by its manufacturer, is not guaranteed or endorsed by the publisher.
